# Editorial: Glycolysis paradigm shift calls for reevaluation of functional brain imaging and pathology analyses

**DOI:** 10.3389/fnins.2023.1215829

**Published:** 2023-06-27

**Authors:** Avital Schurr

**Affiliations:** Department of Anesthesiology and Perioperative Medicine, School of Medicine, University of Louisville, Louisville, KY, United States

**Keywords:** brain functions, energy metabolism, glycolysis, glycogen, lactate

Over the past 35 years our understanding of the role of lactate in brain energy metabolism has greatly increased. Not only the concept of lactate being a waste product of anaerobic glycolysis has been debunked, but this monocarboxylate has been shown to be involved in several functions of the central nervous system (CNS). Since many of the methodologies for measuring cerebral metabolic rates were developed over decades, where glucose and oxygen were assumed to be the two main substrates necessary for the production of adenosine triphosphate (ATP), none are taking into account the role that lactate may plays in this process. Even the most advanced methodologies, such as functional brain imaging, do not include in their measurements and calculations the contribution of lactate to the production of cerebral ATP (Schurr, 2018). This Research Topic aims at highlighting recent studies that support the call to reevaluate the measurements and calculations of cerebral energy metabolic rates in health and disease. In the first Review Article, Rich et al. summarize our knowledge and understanding of the role of astrocytic glycogen and its conversion to lactate in energy metabolism that supports neuronal functions. More specifically, they focus on the rodent optic nerve, where glycogen (lactate) supports axonal metabolism during aglycemia, hypoglycemia or during periods of high energy demands under normoglycemia and the vital role lactate plays when hippocampal neurons are supplemented with it during memory formation. In their Mini Review, Deitmer et al. examine the contributions of astrocytes to energy metabolism and pH homeostasis. They explore the roles of lactate, H^+^, monocarboxylate transporters (MCTs) and carbonic anhydrases (CAs) in physiological processes of energy dynamics in astrocytes and the transfer of energetic substrates to neurons. The third contribution, a Perspective Article by Goodwin et al., deals with lactate-protected hypoglycemia (LPH), a concept originally proposed to target tumors by lowering glucose, while simultaneously increasing lactate. The authors suggest that by exploiting and targeting lactate transport and metabolism novel methods could be developed to treat pathologies of the CNS. Both experimental and observational evidence are discussed that provide direction for developing therapies based on these concepts.

## Author contributions

The author confirms being the sole contributor of this work and has approved it for publication.

## References

[B1] Schurr A. (2018). Glycolysis paradigm shift dictates a reevaluation of glucose and oxygen metabolic rates of activated neural tissue. Front. Neurosci. 12, 700. 10.3389/fnins.2018.00700 30364172 PMC6192285

